# Ethanolic Extract of Kinkeliba (*Combretum micranthum*), Rich in Phenolic Compounds Mitigates DSS-Induced Ulcerative Colitis in C57BL/6 Mice via Antioxidation and Microbiota Regulation

**DOI:** 10.3390/ijms262311299

**Published:** 2025-11-22

**Authors:** Ibrahima Mamadou Sall, Dan Cristian Vodnar, Gheorghe Adrian Martău, Meriem Aziez, Alina Diana Haşaş, Dragoş Hodor, Alexandru Flaviu Tăbăran

**Affiliations:** 1 Department of Anatomic Pathology, Faculty of Veterinary Medicine, University of Agricultural Sciences and Veterinary Medicine of Cluj-Napoca, 400372 Cluj-Napoca, Romania; ibrahima.sall@student.usamvcluj.ro (I.M.S.);; 2 Department of Food Science, Faculty of Food Science and Technology, University of Agricultural Sciences and Veterinary Medicine Cluj-Napoca, 400372 Cluj-Napoca, Romania; 3 Laboratory of Plant Biotechnology and Ethnobotany, Faculty of Nature and Life Sciences, University of Bejaia, Bejaia 06000, Algeria; 4 Department of Pathophysiology, Faculty of Veterinary Medicine, University of Agricultural Sciences and Veterinary Medicine of Cluj-Napoca, 400372 Cluj-Napoca, Romania

**Keywords:** *Combretum micranthum* G. Don, inflammatory bowel disease, DSS-induced colitis, phenolic compounds, HPLC-DAD-ESI-MS

## Abstract

Inflammatory bowel diseases (IBDs), including Crohn’s disease and ulcerative colitis, are chronic inflammatory disorders of the gastrointestinal tract, and current therapies are limited by adverse side effects. *Combretum micranthum* G. Don (kinkeliba), a medicinal plant traditionally used in West Africa, has been reported to possess pharmacological activities and a favorable safety profile. In this study, an ethanolic extract of *Combretum micranthum* (EECM) was characterized using HPLC-DAD-ESI-MS to identify its phenolic constituents. Acute colitis was induced in C57BL/6 mice using 3% DSS, while EECM (100 and 200 mg/kg) was administered orally for seven days. Disease Activity Index was monitored daily, and colonic injuries were evaluated through macroscopic and histological analyses, as well as hematological and biochemical assessments. In vitro, EECM contained 293.54 mg/g of total phenolic compounds and showed strong antioxidant activity in DPPH, ABTS, and FRAP assays. Furthermore, the extract exhibited antibacterial activity against *Bacillus subtilis*, *Staphylococcus aureus*, *Salmonella enterica*, and *Streptococcus pyogenes* at various concentrations. In contrast, *Enterococcus faecalis*, *Escherichia coli*, and *Pseudomonas aeruginosa* were not affected at the tested concentrations. No antifungal activity was detected against the filamentous fungus *Aspergillus brasiliensis* and the yeasts *Saccharomyces cerevisiae*, *Candida parapsilosis*, and *Candida albicans*. In vivo, EECM alleviated the clinical signs of colitis, reduced histological damage, and modulated hematological and biochemical parameters. Overall, EECM exhibited significant antioxidant and anti-inflammatory activities and may represent a promising natural candidate for IBD management. Further investigations into chronic experimental models are necessary to establish their therapeutic relevance.

## 1. Introduction

Chronic inflammatory bowel diseases (IBDs) encompass two primary pathological forms: Crohn’s disease (CD) and ulcerative colitis (UC), which involve persistent inflammation of the gastrointestinal tract [1,2]. UC, inflammation is typically confined to the rectum and colon, while CD can affect any segment of the digestive tract. Clinically, both conditions share common clinical manifestations including abdominal pain, diarrhea, mild fever, and weight loss. These diseases typically exhibit a relapsing-remitting pattern, alternating between periods of remission and exacerbation [3,4,5]. The pathogenesis of IBD is multifactorial and remains incompletely understood, involving interactions among genetic predispositions, environmental factors, immune system dysfunction, and alterations in the gut microbiota. A differential involvement of T helper cells is observed, with Th1 responses predominating in CD and Th2 responses contributing more to UC pathophysiology [6]. However, the activation of Th17 cells and the imbalance between Th17 and regulatory T (Treg) cells have recently been identified as critical contributors to the development of intestinal inflammation [7]. These distinct T helper cell profiles contribute to the chronicity and tissue-specific manifestations of IBD, shaping the immune microenvironment and influencing disease severity. This immunological heterogeneity underscores the complexity of IBD pathophysiology and the need for targeted therapeutic strategies [8,9,10].

Management of IBD predominantly involves anti-inflammatory drugs, including 5-aminosalicylates (5-ASA), corticosteroids, selected antibiotics, and targeted biologics such as anti-TNF-α agents, alongside other immunosuppressive or immunomodulatory therapies. Although effective, these therapies often exhibit variable patient responses and are associated with adverse effects and potential long-term complications [8,9]. Achieving sustained remission remains challenging, as current interventions may not fully control chronic intestinal inflammation. Consequently, there is a pressing need for treatments that selectively attenuate gut inflammation without broadly suppressing systemic immunity. This has prompted increasing interest in bioactive compounds from natural sources, which may provide targeted anti-inflammatory effects with improved safety. These agents are particularly attractive due to their potential to modulate specific immune pathways while minimizing systemic toxicity [5,10,11,12]. A comprehensive understanding of IBD pathophysiology, combined with novel therapeutic strategies, is essential to optimize clinical management and achieve durable remission.

Moreover, an emerging field of interest focuses on bacterial outer membrane vesicles produced by Gram-negative bacteria of the intestinal microbiota. These OMVs can act as vectors for microbe–host communication, carrying lipopolysaccharides, membrane proteins, and nucleic acids. Some studies have shown that OMVs derived from adherent-invasive Escherichia coli strains promote intestinal inflammation and disrupt epithelial barrier integrity, notably by stimulating the expression of TLRs and the production of pro-inflammatory cytokines (IL-8, TNF-α) [13,14]. Other studies indicate that under inflammatory conditions, certain bacterial membrane vesicles can even modulate the microbiota to restore intestinal anaerobiosis, reduce TNF-α production, and improve symptoms in mice [15]. Thus, OMVs appear to be key players in mediating microbial inflammatory signals [16].

*Combretum micranthum* G. Don, locally known as kinkéliba, belongs to the Combretaceae family, which comprises about 20 genera and 600 species [16,17,18,19]. The genus *Combretum* itself includes approximately 250 species distributed mainly in tropical and subtropical regions [20]. *C. micranthum* is a shrub reaching 4–5 m in height, adapted to dry climates, and widely present in the Sahelian and Sudanian regions of West Africa [21]. In traditional medicine, its leaves are commonly prepared as infusions or decoctions and have been employed for the prevention and treatment of various disorders, including diabetes, hypertension, bronchitis, fever, malaria, and intestinal complaints [22,23,24]. These long-standing ethnopharmacological practices underscore its importance as a medicinal resource in West African communities.

Phytochemical investigations have identified a wide range of bioactive constituents in *C. micranthum*, including proteins, fatty acids, amino acids, carbohydrates, vitamins, minerals, flavonoids, phenolic acids, sterols, and alkaloids [25,26]. These compounds are associated with multiple pharmacological activities, notably antioxidant, antimicrobial, anti-inflammatory, hypoglycemic, hepatoprotective, nephroprotective, immunostimulant, and antimalarial properties [27,28,29,30,31]. Several experimental studies have confirmed antibacterial activity against diverse Gram-positive and Gram-negative species [32,33,34,35,36,37], antifungal effects against *Candida albicans* [36], antiviral activity against herpes simplex viruses [17] antimalarial effects against *Plasmodium falciparum* [37,38], and antidiabetic properties comparable to glibenclamide in murine models [28]. Collectively, these findings provide a strong scientific rationale for further exploration of its therapeutic applications.

Recent research highlights those certain anti-inflammatory agents, particularly quercitrin, exert significant anti-inflammatory effects in experimental models of colitis. For example, Camuesco et al. demonstrated that quercitrin exhibits intestinal anti-inflammatory activity in experimental colitis models by modulating the local immune response [18]. Other recent studies support this role, showing that quercetin alleviates DSS-induced colon inflammation by inhibiting NF-κB pathway activation and decreasing pro-inflammatory cytokines (e.g., IL-6, TNF-α) [19,20,21]. These studies suggest that flavonoids may act at multiple levels—as antioxidants, immunomodulators, and protectors of the epithelial barrier.

To the best of our knowledge, no study has yet evaluated the therapeutic effects of *C. micranthum* in DSS-induced colitis, despite ethnobotanical reports supporting its traditional use for intestinal inflammation [22,23]. This gap in the literature highlights the need for experimental validation of its potential in inflammatory bowel disease. Therefore, the present study aimed to provide a comprehensive assessment of ethanolic extract *C. micranthum* by characterizing its phytochemical profile and evaluating its antioxidant and antimicrobial properties in vitro, as well as its protective effects in vivo in a DSS-induced acute colitis model in C57BL/6 mice.

## 2. Results

### 2.1. Phenolic Composition of the Ethanolic Extract of Combretum micranthum

**Figure 1:**
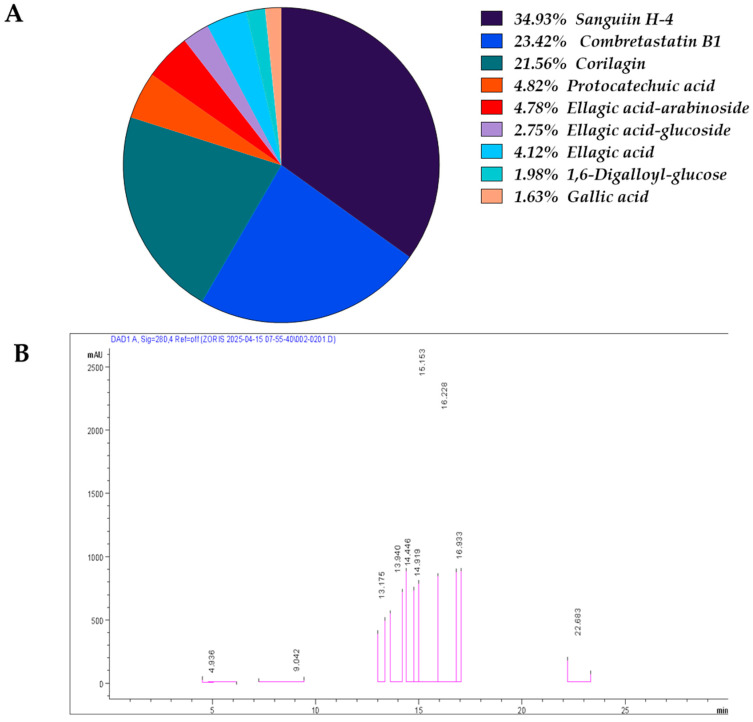
Phenolic composition of the ethanolic extract of EECM: (**A**) Relative distribution (%) of the phenolic compounds identified in the EECM, represented as a circular chart illustrating the proportion of major constituents (Sanguiin H-4, Corilagin, and Combretastatin B1) and minor ones (Protocatechuic acid, Ellagic acid-arabinoside, Ellagic acid-glucoside, Ellagic acid, Gallic acid, and 1,6-Digalloyl-glucose). (**B**) Representative DAD chromatogram obtained by HPLC-DAD-ESI-MS analysis, showing the retention times and relative peak intensities of the identified compounds. The major peaks, with retention times corresponding to Combretastatin B1 (22.69 min), Corilagin (16.22 min), and Sanguiin H-4 (15.40 min), together account for approximately 79.88% of the total phenolic compounds. The minor peaks, with retention times corresponding to Ellagic acid (4.4 min), Protocatechuic acid (9.09 min), 1,6-Digalloyl-glucose (13.16 min), Ellagic acid-arabinoside (13.93 min), Ellagic acid-glucoside (14.43 min), and Ellagic acid (16.63 min), represent the remaining 20.12%.

The overall phenolic content of the ethanolic extract of *Combretum micranthum* (EECM) was determined to be 293.54 mg/g of extract, confirming a rich polyphenolic profile. The detailed chromatographic profile (Figure 1A) and quantitative data of the identified compounds are presented in Figure 1B and Supplementary Materials Table S1 To facilitate visualization, the relative contribution of each phenolic compound is illustrated in Figure 1A, showing the proportion of major and minor constituents in a circular plot. Major constituents were represented by Sanguiin H-4 (34.92%), Corilagin (21.55%), and Combretastatin B1 (23.41%), together accounting for approximately 79.88% of total phenolics. Minor components included Protocatechuic acid (4.82%), Ellagic acid-arabinoside (4.78%), Ellagic acid-glucoside (2.75%), Ellagic acid (4.12%), Gallic acid (1.63%), and 1,6-Digalloyl-glucose (1.98%), collectively representing the remaining 20.12% of the phenolic fraction. These results demonstrate that the extract is predominantly composed of ellagitannins and stilbenes, with hydroxybenzoic acids present in lower proportions, providing a diverse polyphenolic composition likely contributing to its bioactive properties.

### 2.2. In Vitro Biological Activities

#### 2.2.1. Antioxidant Activity

The antioxidant potential of the ethanolic extract of *Combretum micranthum* (EECM) was evaluated using DPPH, ABTS, and FRAP radical scavenging assays at a concentration of 25 mg/mL (Table 1). The extract exhibited notable antioxidant activity, with DPPH scavenging of 135.86 ± 2.35 mmol TE/g, ABTS activity of 233.72 ± 2.81, and FRAP reducing power of 428.86 ± 145.09 mmol TE/g. These results highlight the extract’s ability to neutralize free radicals and reduce ferric ions at this tested concentration.

#### 2.2.2. Antimicrobial Activity

The antimicrobial potential of the ethanolic extract of *Combretum micranthum* (EECM) was evaluated against Gram-positive and Gram-negative bacteria, as well as selected yeast and filamentous fungi, using a resazurin-based microtiter assay. Positive controls were gentamicin (for bacteria) and ketoconazole (for fungi), while sterile water was used as the negative control. Due to the limited solubility of the extract in aqueous medium, the maximum biologically available concentration was approximately 25 mg/mL. Consequently, the reported minimum inhibitory concentrations (MICs) reflect the soluble fraction of the extract rather than the total amount of powder added.

As summarized in Table 2, the extract exhibited selective antibacterial activity: *Staphylococcus aureus* is inhibited at 25,000 µg/mL, *Bacillus subtilis* and *Streptococcus pyogenes* are inhibited at 12,500 µg/mL, while *Enterococcus faecalis* was not inhibited. Among Gram-negative bacteria, only *Salmonella enterica* showed limited susceptibility 1250 µg/mL), whereas *Escherichia coli* and *Pseudomonas aeruginosa* were unaffected.

No inhibitory effect was observed against the tested yeast (*Saccharomyces cerevisiae*, *Candida parapsilosis*, *Candida albicans*) or the filamentous fungus *Aspergillus brasiliensis* (Table 3).

These results indicate that the extract possesses selective antibacterial properties, particularly against Gram-positive bacteria. The solubility-limited nature of the assay highlights that the effective antimicrobial activity corresponds to the fraction of extract that is actually dissolved.

### 2.3. In Vivo Study

#### 2.3.1. Clinical and Morphological Parameters of the Effect of *Combretum micranthum* on the Severity of Colitis

**Figure 2:**
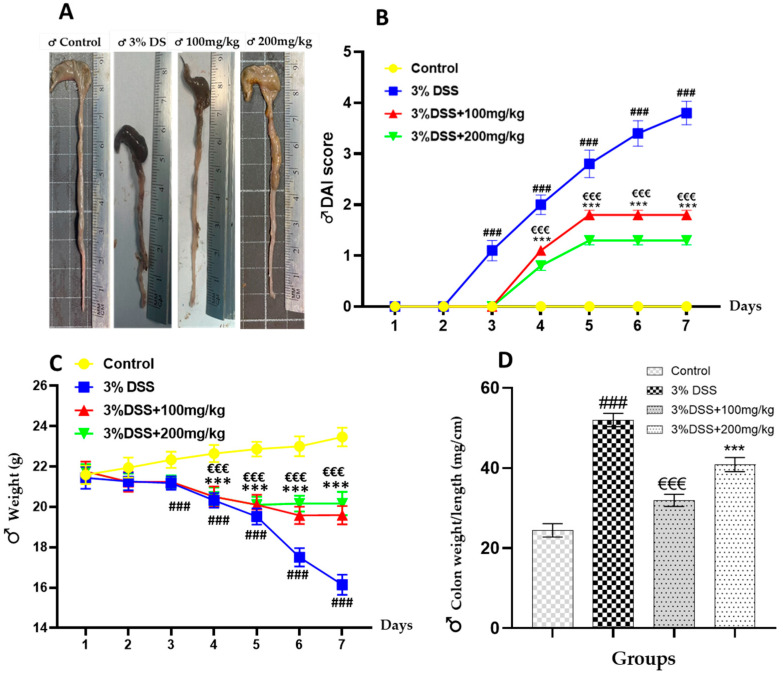
Effect of EECM (100 and 200 mg/kg) administration on clinical and morphological parameters in DSS-induced colitis in male C57BL/6 mice. (**A**) Representative macroscopic appearance of colons from each experimental group, illustrating colon shortening and thickening in DSS-treated mice, and partial recovery in EECM-treated groups. (**B**) Disease Activity Index (DAI) score showing a significant (*p* < 0.001) increase in the DSS group from day 3, which was progressively reduced by EECM administration. (**C**) Body weight, a significant decrease (*p* < 0.001) was observed from day 3 in the 3% DSS colitic group compared to the control group, while a mild improvement was noted in mice treated with EECM. (**D**) Colon weight/length ratio reflecting the severity of colonic inflammation. The ratio was significantly elevated in DSS-induced colitic males (51.96 ± 1.68) compared with controls (24.41 ± 1.67), while treatment with EECM markedly reduced this ratio (31.89 ± 1.53 and 40.85 ± 1.77 for 100 and 200 mg/kg, respectively). Data are expressed as mean ± SD. Statistical significance was determined by one-way ANOVA followed by Tukey’s post hoc test. Comparisons: (### *p* < 0.001) 3% DSS vs. control; (€€€ *p* < 0.001) 3% DSS + 100 mg/kg and (*** *p* < 0.001) 3% DSS + 200 mg/kg vs. 3% DSS group.

**Figure 3:**
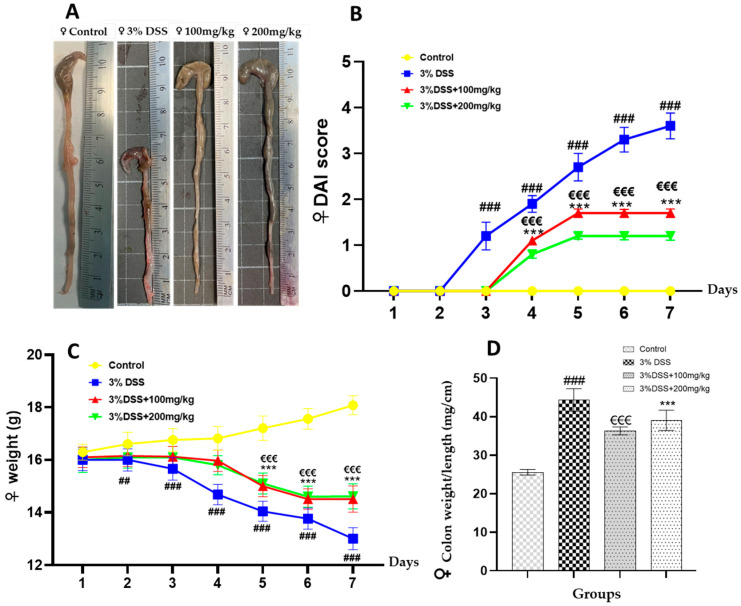
Effect of EECM administration (100 and 200 mg/kg) on clinical and morphological parameters in DSS-induced colitis in female C57BL/6 mice. (**A**) Representative macroscopic images of colons from each experimental group, illustrating the protective effect of EECM against DSS-induced lesions. (**B**) Disease Activity Index (DAI), showing a progressive increase in the score in the DSS group and a dose-dependent reduction following EECM administration. (**C**) Body weight, a significant decrease (*p* < 0.001) was observed from day 3 in the 3% DSS colitic group compared to the control group, while a mild improvement was noted in mice treated with EECM. (**D**) Colon weight/length ratio, markedly increased in the DSS group (44.30 ± 2.95) compared to the control group (25.54 ± 0.73), and significantly reduced after EECM treatment. Data are expressed as mean ± standard deviation (SD). Statistical analysis was performed using one-way ANOVA followed by Tukey’s post hoc test. Comparisons: (## *p* < 0.01, ### *p* < 0.001) 3% DSS group vs. control; (€€€ *p* < 0.001) 3% DSS + 100 mg/kg and (*** *p* < 0.001) 3% DSS + 200 mg/kg vs. 3% DSS group.

This study assessed the antioxidant and anti-inflammatory properties of orally administered EECM using a DSS-induced colitis model. A significant increase (*p* < 0.001) in the colon weight/length ratio was observed in the male colitic group (51.96 ± 1.68) compared to the control group of non-colitic male mice (24.41 ± 1.67) (Figure 2D). Similarly, in female mice, a significant increase (*p* < 0.001) in the weight/length ratio was noted in the colitic group (44.30 ± 2.95) compared to the control group of untreated females (25.54 ± 0.73) (Figure 3D). The groups administered 100 and 200 mg/kg EECM exhibited values of (31.89 ± 1.53) and (40.85 ± 1.77), respectively, which significantly reduced the colon weight/length ratio compared to the colitic group (44.30 ± 2.95) in males (Figure 2D). A similar trend was observed in females (Figure 3D). The colon weight/length ratio serves as a crucial indicator of colonic inflammation. In this study, mice in the colitic group demonstrated reduced colon length and increased colon weight, resulting in an elevated ratio, as ulcerative colitis leads to colon shortening. Administration of EECM resulted in a marked decrease in the colon weight/length ratio, confirming reduced colitis severity.

Moreover, a significant (*p* < 0.001) and progressive increase in the Disease Activity Index (DAI) was observed from day 3 of colitis induction in the 3% DSS colitic group compared to the control group in male mice (Figure 2B), and a similar trend was observed in females (Figure 3B). A significant decrease in body weight (*p* < 0.001) was also noted from day 3 in the 3% DSS colitic group compared to the control group in males (Figure 2C), in females (Figure 3C). However, administration of two doses (100 and 200 mg/kg) of EECM resulted in a progressive reduction in DAI starting from day 5, compared to the control group (Figure 2B), with a moderately similar trend observed in females (Figure 3B). A significant decrease in body weight was also observed with both doses (100 and 200 mg/kg, *p* < 0.001), relative to the colitic group in males (Figure 2C), with a similar trend observed in females (Figure 3C).

#### 2.3.2. Hematological and Biochemical Analysis of Male C57BL/6

In order to further explore the therapeutic potential of EECM, we conducted an evaluation of 18 hematological markers and 13 key biochemical markers. Notably, six hematological markers and five biochemical markers showed significant improvement following EECM administration.

**Figure 4:**
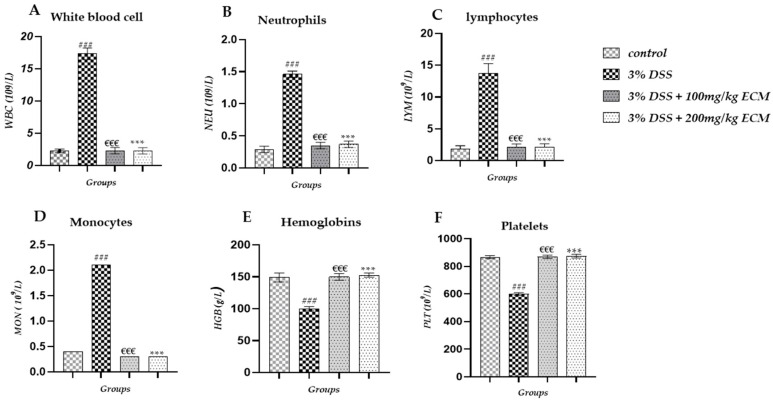
Effect of EECM on hematological markers in DSS-induced colitis in male mice. (**A**) white blood cell (WBC) levels, (**B**) Neutrophils (NEU) levels, (**C**) Lymphocytes (LYM) levels, (**D**) Monocytes (MON) levels, (**E**) Hemoglobins (HGB) levels and (**F**) Platelet (PLT) count. Administration of 3% DSS significantly increased WBC, NEU, LYM, and MON, and reduced HGB levels while increasing PLT count compared to the control group. EECM (100 and 200 mg/kg) significantly corrected these alterations, indicating a systemic anti-inflammatory and protective effect. (### *p* < 0.001) 3% DSS compared to control group. Statistical significance was determined using one-way ANOVA followed by Tukey’s post hoc test. Comparisons were made as follows: (### *p* < 0.001) 3% DSS group vs. control group, (€€€ *p* < 0.001) 3% DSS + 100 mg/kg and (*** *p* < 0.001) 3% DSS + 200 mg/kg vs. 3% DSS group.

Hematologic analysis of males revealed a significant increase in white blood cell (WBC) levels in the 3% DSS group (17.34 ± 0.9 × 10^9^/L; *p* < 0.001) compared to the control group (2.26 ± 0.1 × 10^9^/L). In contrast, significantly reduced WBC levels were observed in the 3% DSS + 100 mg/kg (0.35 ± 0.01 × 10^9^/L *p* < 0.01) and 3% DSS + 200 mg/kg (2.27 ± 0.1 × 10^9^/LP < 0.001) groups compared to the 3% DSS group, as shown in Figure 4A. A significant increase in neutrophil levels (NEU) was also observed in the 3% DSS group (1.46 ± 0.02 × 10^9^/L; *p* < 0.01) compared to the control group (0.29 ± 0.01 × 10^9^/L). EECM administration significantly attenuated this elevation in the 3% DSS + 100 mg/kg (0.35 ± 0.01 × 10^9^/L; *p* < 0.01) and 3% DSS + 200 mg/kg (0.37 ± 0.01 × 10^9^/L; *p* < 0.01) groups compared to the 3% DSS group, as shown in Figure 4B. DSS instillation led to a significant increase in lymphocytes (LYM) in the 3% DSS group (13.75 ± 1.01 × 10^9^/L; *p* < 0.001) compared to the control group (1.82 ± 0.01 × 10^9^/L). This increase was significantly reduced in colitis mice administered with EECM, with values of 3%DSS + 100 mg/kg (2.12 ± 0.04 × 10^9^/L, *p* < 0.001) and 3%DSS + 200 mg/kg (2.15 ± 0.03 × 10^9^/L, *p* < 0.001) compared to 3% DSS group, as shown in Figure 4C. We also observed a significant increase in monocytes (MON) in the 3% DSS group (2.10 ± 0 × 10^9^/L (*p* < 0.001) compared to the control group (0.04 ± 0 × 10^9^/L). This increase was significantly reduced in colitis mice administered with 3%DSS + 100 mg/kg (0.03 ± 0 × 10^9^/L, *p* < 0.01) and 3%DSS + 200 mg/kg (0.03 ± 0 × 10^9^/L, *p* < 0.01) compared to the 3% DSS group, as shown in Figure 4D. A significant decrease in hemoglobin (HGB) levels was observed in the 3% DSS group (100 ± 3.4 g/L, *p* < 0.001) compared to the control group (149 ± 7.1 g/L). However, EECM administration significantly restored HGB levels in the 3%DSS + 100 mg/kg (150 ± 5.2 g/L, €€€ *p* < 0.001) and 3%DSS + 200 mg/kg (153 ± 3.3 g/L, *p* < 0.001) compared to the 3% DSS group, as shown in Figure 4E. We also observed a significant increase in platelet (PLT) count in the 3% DSS group (600 ± 10.3 × 10^9^/L, *p* < 0.001) compared to the control group (867 ± 11.2 × 10^9^/L). However, EECM administration significantly restored PLT levels in the 3%DSS + 100 mg/kg (870 ± 12.2 × 10^9^/L, *p* < 0.001) and 3%DSS + 200 mg/kg (875 ± 13.2 × 10^9^/L, *p* < 0.001) compared to the 3% DSS group, as shown in Figure 4F. More detailed information on the hematological analysis in male mice can be found in Supplementary Materials Table S2.

**Figure 5:**
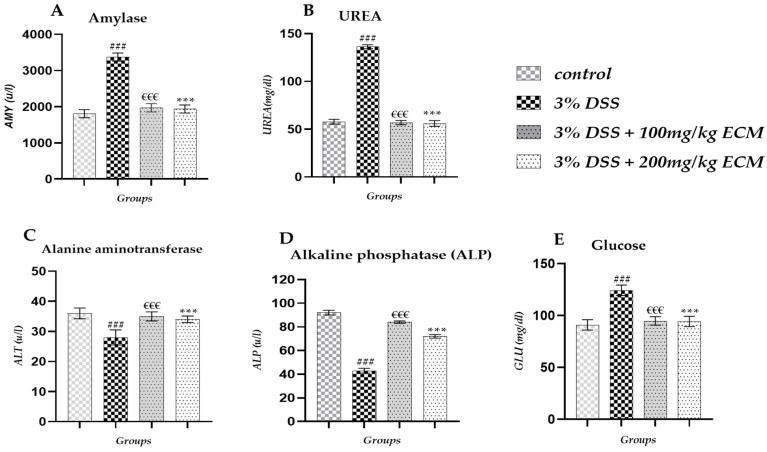
Effect of EECM on biochemical markers in DSS-induced colitis in male mice. (**A**) Amylose (AMY) levels, (**B**) Urea (UREA) levels, (**C**) Alanine aminotransferase (ALT) levels, (**D**) Alkaline phosphate (ALP) levels and (**E**) Glucose (GLU) levels Induction of colitis by DSS resulted in significant alterations in biochemical markers, including an increase in AMY, UREA, and GLU, as well as a decrease in ALT and ALP compared to the control group. Administration of EECM at two doses (100 and 200 mg/kg) significantly corrected these disturbances, reflecting an improvement in hepatic and metabolic function and a systemic protective effect. Data are expressed as mean ± standard deviation (SD). Statistical analysis was performed using one-way ANOVA followed by Tukey’s post hoc test. Comparisons were made as follows: (### *p* < 0.001) 3% DSS vs. control. (€€€ *p* < 0.001) 3% DSS + 100 mg/kg and (*** *p* < 0.001) 3% DSS + 200 mg/kg vs. 3% DSS group.

Biochemical analysis of males revealed that DSS instillation led to a significant increase in amylase (AMY) levels in the 3% DSS group (3374 ± 11.10 u/L; *p* < 0.001) compared to the control group (1807 ± 11.75 u/L). Administration of EECM to colitis mice led to a significant reduction in AMY levels in both tested EECM doses (100 and 200 mg/kg) 3%DSS + 100 mg/kg (1971 ± 15.15 u/L; *p* < 0.001) and 3%DSS + 200 mg/kg (1937 ± 13.15 u/L; *p* < 0.001) compared to in the group 3% DSS as shown in Figure 5A. Induction of colitis by DSS led to a significant increase in urea (UREA) levels in the 3% DSS group (136.34 ± 2.1 mg/dL; *p* < 0.001) compared to that in the control group (57.83 ± 2.5 mg/dL). Administration of EECM to colitis mice resulted in a significant reduction in urea levels in the 3% DSS + 100 mg/kg group (56.88 ± 2.2 mg/dL; *p* < 0.001) and the 3% DSS + 200 mg/kg group (56.03 ± 3 mg/dL; *p* < 0.001) compared to the 3% DSS group, as shown in Figure 5B. We observed a significant increase in glucose levels (GLU) in the 3% DSS group (124.41 ± 2 mg/dL; (*p* < 0.001) compared to the control group (91.02 ± 1 mg/dL). Administration of EECM to colitis mice resulted in a significant reduction in glucose levels in the 3% DSS + 100 mg/kg group (94.91 ± 2 mg/dL; *p* < 0.001) and the 3% DSS + 200 mg/kg group (94.42 ± 1.5 mg/dL; *p* < 0.001) compared to the 3% DSS group, as shown in Figure 5C. A significant decrease in alkaline phosphatase (ALP) levels was observed in the 3% DSS group (43 ± 2 u/L; *p* < 0.001) compared to the control group (92 ± 2 u/L). However, EECM treatment significantly restored ALP levels in the 3% DSS + 100 mg/kg group (84 ± 1 u/L; *p* < 0.001) and the 3% DSS + 200 mg/kg group (72 ± 1.5 u/L; *p* < 0.001) compared to the group 3% DSS as shown in Figure 5D. Alanine aminotransferase levels (ALT) were also statistically significant at 3% DSS (28 ± 2.5 g/uL; *p* < 0.001) compared to the control group (36 ± 1.8 uL). However, EECM administration significantly restored ALP levels in the 3% DSS + 100 mg/kg (35 ± 1.5 u/L; *p* < 0.001) and the 3% DSS + 200 mg/kg (34 ± 1.1 u/L; *p* < 0.001) groups compared to the group 3% DSS as shown in Figure 5E. More detailed information on the biochemical analysis in Male mice can be found in Supplementary Materials Table S2.

#### 2.3.3. Analysis Hematological and Biochemical of Females Mice in C57BL/6

Similarly, in female C57BL/6 mice, 18 hematological parameters and 13 biochemical parameters were assessed. Among these, five hematological and five biochemical markers showed significant improvement following EECM administration.

**Figure 6:**
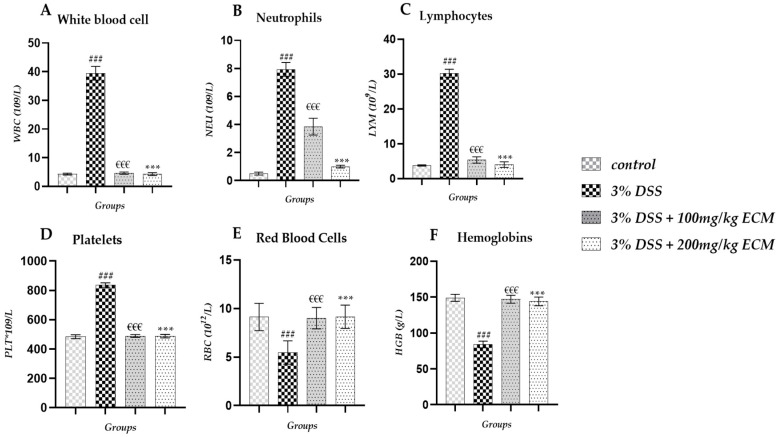
Effect of EECM on hematological parameters in DSS-induced colitis in female mice. In female C57BL/6 mice, induction of colitis by DSS caused significant alterations in hematological parameters, with a marked increase in (**A**) WBC, (**B**) NEU, (**C**) LYM, and (**D**) PLT, as well as a decrease in (**E**) RBC and (**F**) HGB compared to the control group. Administration of EECM at two doses (100 and 200 mg/kg) significantly corrected these disturbances, reflecting a protective effect of EECM on inflammation and hematological function. Data are expressed as mean ± standard deviation (SD). Statistical analysis was performed using one-way ANOVA followed by Tukey’s post hoc test. Comparisons were made as follows: (### *p* < 0.001) 3% DSS compared to control group. (€€€ *p* < 0.001) 3% DSS + 100 mg/kg and (*** *p* < 0.001) 3% DSS + 200 mg/kg compared to 3% DSS group.

Hematologic analysis of female mice revealed a significant increase in white blood cell (WBC) levels in the 3% DSS group (39.43 ± 2.4 × 10^9^/L; *p* < 0.001) compared to the control group (4.29 ± 0.3 × 10^9^/L). Administration of EECM to colitis mice resulted in a significant reduction in WBC levels in the 3% DSS + 100 mg/kg group (4.62 ± 0.4 × 10^9^/L; *p* < 0.001) and the 3% DSS + 200 mg/kg group (4.28 ± 0.4 × 10^9^/L; *p* < 0.001) compared to the 3% DSS group, as shown in Figure 6A. Neutrophil (NEU) levels showed a statistically significant increase in the 3% DSS group (7.93 ± 0.1 × 10^9^/L; *p* < 0, 001) compared to the control group (0.50 ± 0 × 10^9^/L). Administration of EECM to colitis mice resulted in a significant reduction in NEU levels in the 3% DSS + 100 mg/kg group (3.85 ± 0.6 × 10^9^/L; *p* < 0.001) and the 3% DSS + 200 mg/kg group (41.59 ± 0.1 × 10^9^/L; *p* < 0.001) compared to the 3% DSS group, as shown in Figure 6B. We also observed a significant increase in lymphocyte (LYM) levels in the 3% DSS group (30.20 ± 1.2 × 10^9^/L; *p* < 0.001) compared to the control group (3.79 ± 0.2 × 10^9^/L). Administration of EECM to colitis mice resulted in a significant reduction in LYM levels in the 3% DSS + 100 mg/kg group (5.36 ± 0.9 × 10^9^/L; *p* < 0.001) and the 3% DSS + 200 mg/kg group (4.02 ± 0.8 × 10^9^/L; *p* < 0.001) compared to the 3% DSS group, as shown in Figure 6C. A significant increase in platelet (PLT) levels was observed in the 3% DSS group (836 ± 15 × 10^9^/L; *p* < 0.001) compared to the control group (484 ± 13.5 × 10^9^/L). Administration of EECM to colitis mice resulted in a significant reduction in PLT levels in the 3% DSS + 100 mg/kg group (489 ± 10 × 10^9^/L; *p* < 0.001) and the 3% DSS + 200 mg/kg group (488 ± 12 × 10^9^/L; *p* < 0.001) compared to the 3% DSS group, as shown in Figure 6D. Red blood cell (RBC) levels were statistically decreased in the 3% DSS group (5.47 ± 2.2 × 10^12^/L; *p* < 0.001) compared to the control group (9.13 ± 2.1 × 10^12^/L). However, EECM administration significantly restored RBC levels in the groups 3% DSS + 100 mg/kg (9.01 ± 1.1 × 10^12^/L; *p* < 0.001) and the 3% DSS + 200 mg/kg (9.15 ± 1.9 × 10^12^/L; *p* < 0.001) compared to in the group 3% DSS as shown in Figure 6E. A significant decrease was also observed in hemoglobin (HGB) levels in the 3% DSS group (836 ± 15 × 10^9^/L; *p* < 0.001) compared to the control group (484 ± 13.5 × 10^9^/L). However, EECM administration significantly restored HGB levels in the groups 3% DSS + 100 mg/kg (147 ± 5.5 g/L; *p* < 0.001) and the 3% DSS + 200 mg/kg (144 ± 6 g/L; *p* < 0.001) compared to the group 3% DSS as shown in Figure 6F. More detailed information on the hematological analysis in Female mice can be found in Supplementary Materials Table S3.

**Figure 7:**
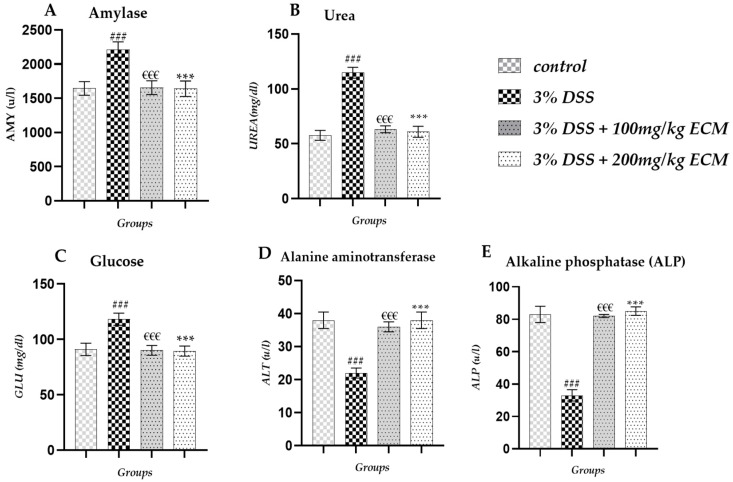
Effect of EECM on biochemical markers in DSS-induced colitis in male mice. In male C57BL/6 mice, induction of colitis by DSS resulted in significant alterations in biochemical markers, with a marked increase in (**A**) AMY, (**B**) UREA, and (**C**) GLU, as well as a decrease in (**D**) ALT and (**E**) ALP compared to the control group. Administration of EECM at two doses (100 and 200 mg/kg) significantly corrected these disturbances, indicating a protective effect of EECM on biochemical function and hepatic and renal metabolism. Data are expressed as mean ± standard deviation (SD). Statistical analysis was performed using one-way ANOVA followed by Tukey’s post hoc test. Comparisons were made as follows: (### *p* < 0.001) 3% DSS compared to control. (€€€ *p* < 0.001) 3% DSS + 100 mg/kg and (*** *p* < 0.001) 3% DSS + 200 mg/kg vs. 3% DSS group.

Biochemical analysis of females revealed a significant increase in amylase levels (AMY) in the 3% DSS group (2212 ± 110.10 u/L; *p* < 0.001) compared to the control group (1643 ± 100 u/L). Administration of EECM to colitis mice led to a significant reduction in AMY levels in both administered EECM doses (100 and 200 mg/kg) 3%DSS + 100 mg/kg (1654 ± 100 u/L; *p* < 0.001) and 3%DSS + 200 mg/kg (1639 ± 114.16 u/L; *p* < 0.001) compared to the group 3% DSS, as shown in Figure 7A. A significant increase in urea (UREA) levels was observed in the 3% DSS group (114.70 ± 5 mg/dL; *p* < 0.001) compared to the control group (57.70 ± 4.7 mg/dL). Administration of EECM to colitis mice resulted in a significant reduction in urea levels in the 3% DSS + 100 mg/kg group (63.23 ± 3.1 mg/dL; *p* < 0.001) and the 3% DSS + 200 mg/kg group (60.90 ± 5 mg/dL; *p* < 0.001) compared to the 3% DSS group, as shown in Figure 7B. Glucose levels (GLU) showed a significant increase in the 3% DSS group (118.21 ± 5.5 mg/dL; (*p* < 0.001) compared to the control group (88.6 ± 5.6 mg/dL). Administration of EECM to colitis mice resulted in a significant reduction in glucose levels in the 3% DSS + 100 mg/kg group (90.10 ± 4.4 mg/dL; *p* < 0.001) and the 3% DSS + 200 mg/kg group (89.42 ± 4.5 mg/dL; *p* < 0.001) compared to the 3% DSS group, as shown in Figure 7C. A significant decrease in alkaline phosphatase (ALP) levels was observed in the 3% DSS group (33 ± 3.5 u/L; *p* < 0.001) compared to the control group (83 ± 5 u/L). However, EECM administration significantly restored ALP levels in the 3% DSS + 100 mg/kg group (82 ± 1 U/L; €€€ *p* < 0.001) and the 3% DSS + 200 mg/kg group (85 ± 2.6 U/L; *p* < 0.001) compared to the group 3% DSS as shown in Figure 7D. Alanine aminotransferase levels (ALT) were also statistically significant at 3% DSS (22 ± 1.5 g/uL; *p* < 0.001) compared to the control group (38 ± 2.5 uL). However, EECM administration significantly restored ALP levels in the groups 3% DSS + 100 mg/kg (36 ± 1.5 u/L; *p* < 0.001) and the 3% DSS + 200 mg/kg (38 ± 2.5 u/L; *p* < 0.001) compared to the group 3% DSS as shown in Figure 7E. More detailed information on the biochemical analysis in Female mice can be found in Supplementary Materials Table S3.

#### 2.3.4. Histological Analysis

**Figure 8:**
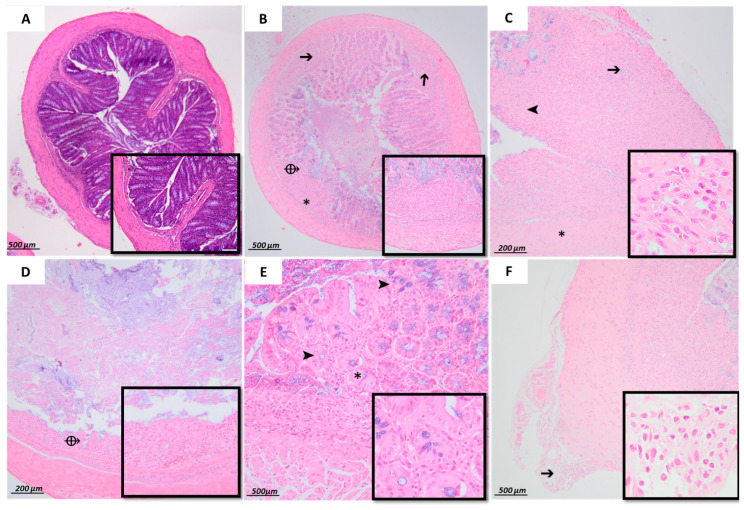
Effect of EECM (100 and 200 mg/kg) on histological alterations in DSS-induced acute colitis in C57BL/6 mice. illustrating (**A**) normal histology in the control group. No significant findings. Histological analysis of acute DSS-induced colitis tissue damage, severe lesions observed in the 3% DSS group (**B**–**F**). illustrating Multifocally, the lamina propria and submucosa are infiltrated by polymorphonuclear cells mixed with mononuclear leukocytes (**B**) (➔) and distended by fibrosis (**B**,**C,E**) (*). Multifocal depletion and complete loss of intestinal crypts is observed (**E**) (➤). The lamina propria and submucosa are diffusely and severely infiltrated by polymorphonuclear cells, with fewer mononuclear leukocytes (➔) and are distended by fibrosis (**C**) (*). The intestinal mucosa is diffusely ulcerated (**B**,**D**) (⟴). Moderate diffuse distention of intestinal crypts and goblet cell depletion is noted (**D**) (➤), and multifocally, the intestinal serosa is infiltrated by polymorphonuclear and mononuclear cells (**F**) (➔). Key: (*) fibrosis in the mucosa or submucosa; (➔) infiltration of polymorphonuclear and mononuclear cells in the mucosa, submucosa and muscular layer; (➤) disruption of crypt and goblet cell depletion; (⟴) ulceration. Representative microphotographs of transverse colon sections stained with hematoxylin and eosin (H&E) at different magnifications (G × 4, G × 10, G × 10, G × 20, and G × 40).

**Figure 9:**
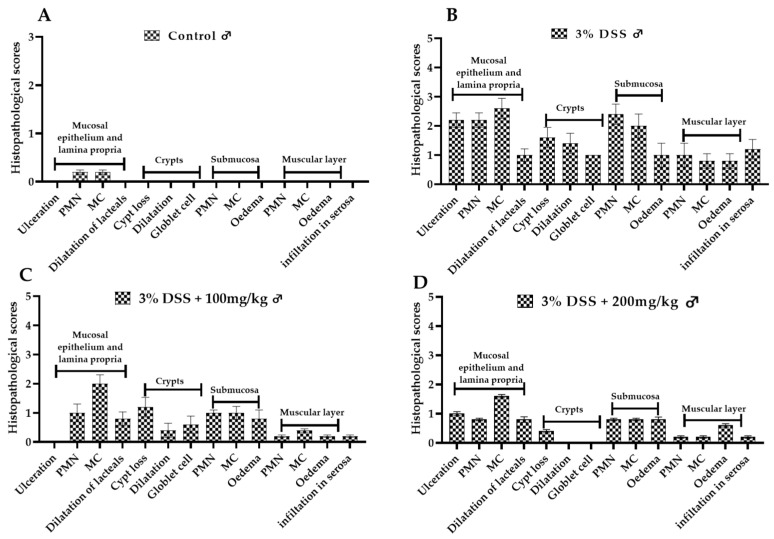
Effect of EECM on histopathological scores in DSS-induced acute colitis in male C57BL/6 mice. Histopathological scores in DSS-induced acute colitis in male C57BL/6 mice. (**A**): control group; (**B**): 3% DSS group; (**C**): 3% DSS + 100 mg/kg EECM and (**D**): DSS + 200 mg/kg EECM). Treatment with EECM at 100 mg/kg and 200 mg/kg (**C**,**D**) reduced the histopathological lesions induced by DSS (**B**). Inflammatory infiltrates in the mucosa and submucosa were decreased, and epithelial structures, including crypts and goblet cells, were better preserved. Scores were graded according to severity: 0 = no significant change, 1 = minimal, 2 = mild, 3 = moderate, 4 = severe.

**Figure 10:**
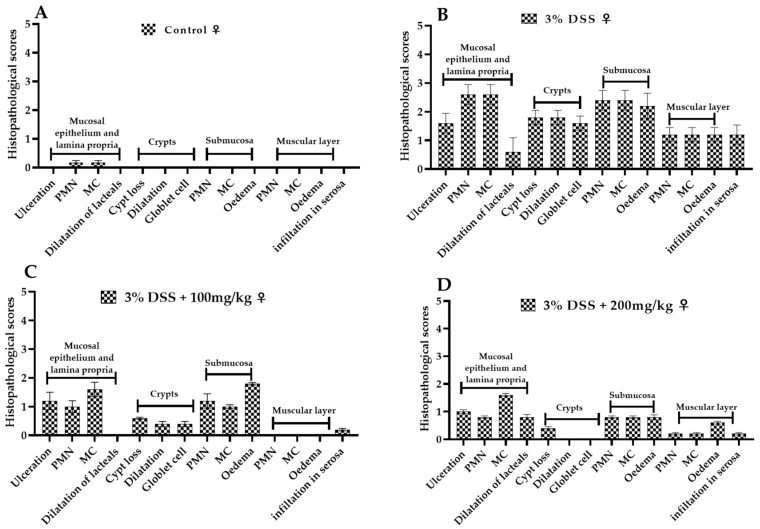
Effect of EECM on histopathological scores in DSS-induced acute colitis in female C57BL/6 mice. Histopathological scores in DSS-induced acute colitis in female C57BL/6 mice. (**A**): control group; (**B**): 3% DSS group; (**C**): 3% DSS + 100 mg/kg EECM and (**D**): DSS + 200 mg/kg EECM). Treatment with EECM at 100 mg/kg and 200 mg/kg (**C**,**D**) reduced the histopathological lesions induced by DSS (**B**). Inflammatory infiltrates in the mucosa and submucosa were decreased, and epithelial structures, including crypts and goblet cells, were better pre-served. Scores were graded according to severity: 0 = no significant change, 1 = minimal, 2 = mild, 3 = moderate, 4 = severe.

Histological analysis further highlighted the severity of acute colonic tissue inflammation induced by DSS and the anti-inflammatory and antioxidant effects of EECM in DSS-induced acute colitis in C57BL/6 mice. Microscopic examination of colonic samples from the untreated colitic group (3% DSS) revealed significant histopathological alterations. Multifocally and diffusely, the lamina propria and submucosa were infiltrated by polymorphonuclear cells mixed with mononuclear leukocytes and showed fibrotic distension. The intestinal mucosa was diffusely ulcerated. Moderate diffuse distention of the intestinal crypts and goblet cell depletion were also observed. Additionally, the intestinal serosa was infiltrated by both polymorphonuclear and mononuclear cells (Figure 8B). However, microscopic examination of samples from the non-colitic group (control group) revealed well-preserved colonic architecture, with intact epithelial structures and no signs of inflammatory infiltration (Figure 8A). Histopathological scores (Figure 9 and Figure 10) showed that EECM administration in DSS-induced acute colitis in C57BL/6 mice resulted in a significant reduction in colitis lesions (Figure 9B and Figure 10B). Improved histological appearance, such as reduced inflammatory infiltrates of the mucosa and submucosa and preservation of epithelial structures, including intestinal crypts and goblet cells, was observed mainly in colitis samples from mice treated with 100 mg/kg and 200 mg/kg EECM in male mice (Figure 9C,D) and female mice (Figure 10C,D) compared to the untreated colitic group 3% DSS in male (Figure 9B) and female mice (Figure 10B). The histological results obtained from the groups of mice administered with EECM extract demonstrated the anti-inflammatory and antioxidant effects of EECM in the intestine. This anti-inflammatory and antioxidant effect was confirmed by a reduction in immune cell infiltration and preservation of the intact crypt structure (Figure 8). More detailed information on the histopathological scores can be found in Supplementary Materials Table S4.

## 3. Discussion

In recent years, our understanding of IBD has significantly advanced, largely owing to the development of well-characterized experimental mouse models [24]. Among these, the DSS-induced colitis murine model has been essential in providing a reproducible and clinically relevant platform to evaluate potential therapeutic agents and dissect disease mechanisms [25]. This model mimics many features of human UC, including clinical symptoms and morphological changes, such as mucosal ulceration, epithelial barrier disruption, and innate immune activation, thereby offering crucial insights into the pathophysiology of IBD, as previously reported [26,27].

In the present study, we aimed to further elucidate the phenolic compound profile of the ethanolic extract of *Combretum micranthum* (EECM) using HPLC-DAD-ESI-MS to clarify the mechanisms by which it exerts its antioxidant and anti-inflammatory effects in a DSS-induced colitis model. Chromatographic analysis of the EECM revealed a total phenolic content of 293.54 mg/g of extract, confirming the plant’s rich polyphenolic profile. This detailed characterization provides a basis for understanding the observed biological effects. Ellagitannins were predominant, mainly represented by Sanguiin H-4 (34.92%) and corilagin (21.55%), alongside the notable stilbene Combretastatin B1 (23.41%), suggesting that these three compounds constitute the major bioactive matrix of the extract. This profile aligns with the findings of [28], who reported significant levels of gallic acid, catechin, and quercetin-3-glucoside in the ethanolic extracts of C. micranthum. Other studies have shown that ellagitannins and stilbenes are strongly associated with antioxidant and anti-inflammatory properties, contributing to the attenuation of oxidative stress and modulation of inflammatory pathways [29,30]. Minor compounds such as protocatechuic acid (4.82%), ellagic acid (4.12%), and gallic acid (1.63%), although present in low proportions, may act synergistically to enhance the overall biological effect of the extract. This hypothesis is consistent with the findings of Gao et al. [31], who demonstrated the protective effect of polyphenols against dextran sulfate sodium (DSS)-induced colitis in mice. The high proportion of ellagitannins and stilbenes identified in EECM may explain the beneficial effects observed on the clinical and histopathological parameters in the DSS-induced colitis model. Studies have shown that Corilagin inhibits the production of TNF-α, IL-1β, IL-6, and NO via the NF-κB pathway and induces HO-1 expression, providing anti-inflammatory and cytoprotective effects [30]. Sanguiin H-4 has also been reported to reduce TLR-4 expression and inflammatory mediator production, thereby regulating the innate immune response [32]. As a bioactive stilbene, Combretastatin B1 may contribute to the regulation of the inflammatory environment and protection of tissues from oxidative damage [33]. Together, the combination of these major and minor phenolic compounds appears to play a key role in reducing leukocyte infiltration and preserving crypt architecture in mice treated with EECM.

The results of in vitro antioxidant assays confirmed that the EECM exhibited significant antioxidant activity. Notably, slightly higher activity was observed in the FRAP assay compared to the ABTS and DPPH assays, highlighting the extract’s ability to effectively scavenge different radical species. Overall, these findings demonstrate that EECM is a rich source of bioactive polyphenols with a strong capacity to neutralize reactive oxygen species (ROS) and mitigate oxidative stress. This suggests a potential synergistic action among the phenolic compounds in EECM, consistent with the findings of Zannou et al. [28]; Welch [34]; and Kpemissi [35] in other experimental models. This property is particularly relevant in DSS-induced ulcerative colitis, where oxidative stress plays a central role in epithelial barrier dysfunction and amplification of the inflammatory response [36].

Clinically, treatment, administrated with EECM at different doses (100 and 200 mg/kg) significantly mitigated body weight loss, colon shortening, and the disease activity index (DAI) while normalizing the colon weight/length ratio. These results indicate that EECM effectively attenuates the clinical manifestations of colitis in mice. They also highlight the antioxidant and anti-inflammatory properties of EECM, capable of rapidly neutralizing oxidative stress and modulating early inflammatory responses, thereby preventing exacerbation of tissue damage in the colon. Previous studies have reported the anti-inflammatory properties of EECM in various pathological contexts [34,35,37,38,39]. Hematological and biochemical analyses provided further confirmed these anti-inflammatory effects in the DSS-induced acute colitis model. The elevation of hematological markers (WBCs, neutrophils, lymphocytes and monocytes) observed in DSS-administered C57BL/6 mice indicates an acute inflammatory response consistent with the pathogenesis of colitis and the DSS-induced immune disruption.

This hematological profile is characteristic of a severe inflammatory state associated with intestinal epithelial barrier breakdown, leading to massive infiltration of immune cells and both local and systemic inflammatory responses [40,41]. Moreover, a significant decrease in hemoglobin and platelet levels was recorded, indicating hematological alterations linked to the intestinal inflammatory process. These changes likely result from blood loss due to colonic hemorrhages and suggest thrombocytopenia, potentially caused by increased platelet consumption at sites of inflammation or impaired hematopoiesis. Together, these changes underscore the severity of colitis and the systemic impact of DSS-induced inflammation [42].

In addition, DSS-treated C57BL/6 mice exhibited elevated levels of biochemical markers, including amylase, urea, and glucose. This was accompanied by decreased levels of alanine aminotransferase and alkaline phosphatase, indicating metabolic and functional alterations directly associated with acute inflammation in the colon. The increase in amylase levels may reflect severe mucosal inflammation and subsequent disruption of tissue integrity, leading to enhanced release of digestive enzymes. Elevated urea levels could result from increased intestinal absorption of nitrogenous waste due to epithelial barrier damage, while the observed hyperglycemia may stem from localized inflammatory responses that disrupt intestinal glucose metabolism [42].

However, administration of EECM at doses of 100 and 200 mg/kg significantly restored the altered hematological parameters, including white blood cells, neutrophils, lymphocytes, monocytes, hemoglobin, and platelets. This normalization suggests attenuation of the systemic inflammatory response and improvement in the hematological status of mice with colitis.

Histological analysis conclusively demonstrated the severity of acute colonic inflammation induced by 3% DSS, highlighting the therapeutic potential of EECM in mitigating intestinal injury. In the colitic group (3% DSS), marked histopathological alterations were observed, including diffuse mucosal ulceration, fibrotic distension, and prominent infiltration of both polymorphonuclear and mononuclear inflammatory cells within the lamina propria, submucosa, and serosa, which are hallmark features of acute colitis-associated inflammatory lesions [36,43]. Administration of EECM at doses of 100 and 200 mg/kg significantly improved colonic histological architecture in both male and female mice. In the 3% DSS + 100 mg/kg and 3% DSS + 200 mg/kg EECM groups, a notable reduction in inflammatory infiltrates was observed, along with partial restoration of crypt structures and an increase in goblet cell numbers, suggesting a protective effect of EECM on the intestinal mucosal barrier. These improvements were supported by significantly lower histopathological scores compared to those in the 3% DSS group.

Clinical parameters revealed the development of colitis in mice, with males showing greater susceptibility than females. This difference was reflected by a more pronounced body weight loss and a significantly higher colon weight-to-length ratio in males. These findings are consistent with several previous studies reporting a higher severity of DSS-induced colitis in male mice, likely due to the protective role of estrogens [44,45,46]. In contrast, no significant sex-dependent differences were observed in response to EECM treatment, as both sexes exhibited a comparable restoration of clinical and histological parameters after treatment. This observation suggests that, unlike certain plant-derived compounds whose effects may vary according to sex [47], EECM exerts a balanced therapeutic effect in both male and female mice.

Many studies have reported that disturbances in the intestinal microbial environment play a critical role in UC pathogenesis. Patients with UC generally exhibit reduced microbial diversity and richness, accompanied by significant alterations in the composition of their gut microbiota [48]. In accordance with previous studies [49,50,51], we observed that the composition of certain intestinal bacterial species, including Bacillus subtilis, Staphylococcus aureus, Salmonella enterica, and Streptococcus pyogenes, was altered to varying degrees. However, a polyphenol-based intervention partially reversed these changes, although the extent of restoration differed among species. In particular, Staphylococcus aureus is recognized as an opportunistic pathogenic bacterium implicated in intestinal dysbiosis [52,53]. Polyphenol treatment significantly reduced the relative abundance of Staphylococcus aureus. This intervention also promoted the secretion of anti-inflammatory mediators by inhibiting the expression of NF-κB–dependent genes, thereby decreasing IL-8 production and chemokine release by macrophages. In parallel, polyphenols attenuated inflammatory responses mediated by effector T lymphocytes while enhancing the expression of anti-inflammatory regulatory T cells (Tregs) in mice [53,54]. Furthermore, certain short-chain fatty acid (SCFA) derivatives produced by microbial metabolism exhibit anti-inflammatory properties and contribute to the strengthening of the intestinal barrier [53].

Additionally, recent studies suggest that polyphenol-rich plant extracts exert indirect anti-inflammatory effects through modulation of the gut microbiota. By promoting the growth of beneficial commensal bacteria (such as Lactobacillus and Bifidobacterium) and inhibiting the proliferation of opportunistic pathogens, these compounds help restore microbial diversity and intestinal barrier integrity, which are critical elements in the pathophysiology of colitis [55,56]. It is therefore plausible that similar microbiota-mediated mechanisms contribute to the protective effects observed with EECM in the DSS-induced colitis model, although this hypothesis still requires direct experimental validation.

The observed therapeutic effects may be attributed to the richness of EECM in bioactive compounds, particularly Sanguiin H-4 and Corilagin, two well-known ellagitannins with potent antioxidant, anti-inflammatory, and immunomodulatory properties. Corilagin, in particular, has been shown in vitro to inhibit the production of TNF-α, IL-1β, IL-6, nitric oxide (NO), and cyclooxygenase-2 (COX-2) via the NF-κB pathway, while inducing the expression of heme oxygenase-1 (HO-1) [57]. Additionally, Corilagin modulates the MAPK and TLR signaling pathways to reduce reactive oxygen species (ROS) and pro-inflammatory cytokines [58]. Similarly, ellagitannins, such as Sanguiin H-4, have been reported to modulate neutrophil-mediated inflammatory responses by decreasing the production of inflammatory mediators and downregulating TLR-4 expression [59].

Beyond its direct antioxidant and anti-inflammatory effects, the therapeutic potential of *C.micranthum* extract may also involve modulation of key signaling pathways and the balance of the gut microbiota. Several phenolic compounds identified in EECM, notably ellagitannins and stilbenes, are known to interfere with inflammatory cascades by inhibiting the activation of NF-κB and MAPK pathways, thereby reducing the transcription of pro-inflammatory cytokines such as TNF-α, IL-6, and IL-1β [8,9,10]. Moreover, these compounds can stimulate the expression of cytoprotective enzymes, including HO-1 and SOD, contributing to the restoration of redox balance and regeneration of the intestinal mucosa [60,61].

Overall, these findings highlight the multifactorial mode of action of *C. micranthum* extract, involving both immune modulation and regulation of the gut microbiota. Nevertheless, further studies are required to better elucidate the precise molecular mechanisms underlying these effects and to assess the long-term efficacy and safety of EECM in chronic colitis models and clinical settings [62].

## 4. Materials and Methods

### 4.1. Plant Material and Extraction Method

Samples of *Combretum micranthum* G. Don were collected in Sélibabi, the capital of the Guidimaka region (15.1585° N, 12.1833° W), located in southern Mauritania. Botanical identification was performed at the Herbarium of the Department of Botany, University of Agricultural and Veterinary Sciences of Cluj-Napoca, Romania (voucher no. 30431). For extract preparation, 20 g of dried *C. micranthum* leaves were macerated in 800 mL of 70% ethanol and stirred for 2 h. The resulting mixture was filtered through a Whatman filter paper and centrifuged at 3000 rpm for 10 min. The filtrate was then concentrated at 50 °C using a rotary evaporator. The dry extract was stored at 4 °C until further.

### 4.2. HPLC-DAD-ESI/MS Profiling of Polyphenolic Compounds

The polyphenolic composition of the ethanolic extract of *Combretum micranthum* (EECM) was characterized by HPLC-DAD-ESI/MS, as reported in our previous study [63] and according to the method described by Călinoiu and Vodnar [64] with minor modifications. Briefly, the extract was filtered through a Chromafil Xtra nylon membrane filter (0.45 µm), and 20 μL was injected into an Agilent 1200 HPLC system (Agilent Technologies, Santa Clara, CA, USA) equipped with a quaternary pump, solvent degasser, autosampler, diode array detector (DAD), and a single quadrupole mass spectrometer (model 6110). Separation was performed using a Kinetex XB-C18 column (5 µm, 4.6 × 150 mm, Phenomenex, Torrance, CA, USA). The mobile phases consisted of (A) water with 0.1% acetic acid and (B) acetonitrile with 0.1% acetic acid, respectively. The gradient elution program was set as follows: 0–2 min, 5% B; 2–18 min, linear increase to 40% B; 18–20 min, increase to 90% B; maintained at 90% B until 24 min; decreased to 5% B at 25 min; and held until 30 min.

The flow rate was 0.5 mL/min, and the column temperature was maintained at 25 ± 0.5 °C. UV spectra were collected over the range of 200–600 nm, and chromatograms were monitored at λ = 280 nm and 340 nm. Mass spectrometric detection was performed in positive electrospray ionization (ESI+) full scan mode with the following settings: capillary voltage 3000 V, drying gas temperature 350 °C, nitrogen flow rate 7 L/min, and scan range *m*/*z* 120–1200. Quantification was performed using external calibration curves generated from authentic standards that showed excellent linearity (R^2^ > 0.99). Data acquisition and processing were performed using the Agilent ChemStation software (Rev B.02.01-SR2).

### 4.3. In Vitro Antioxidant Activity

The antioxidant potential of EECM was evaluated using three radical scavenging assays: 2,2′-azino-bis (3-ethylbenzothiazoline-6-sulfonic acid) (ABTS), 2,2-diphenyl-1-picrylhydrazyl (DPPH), and Ferric-Reducing Antioxidant Power (FRAP) methods, according to the procedures described by [65,66,67] with minor modifications. Three assays were adapted to a microplate format, and Trolox was used as the reference. The antioxidant capacity of the extract was expressed as Trolox equivalents (mmol Trolox/g of sample ) for the DPPH, ABTS and FRAP assays. All experiments were performed in triplicate (*n* = 3).

#### 4.3.1. ABTS Radical Scavenging Assay

The extract (20 µL) was mixed with 170 µL of diluted ABTS^+^ solution and incubated for 6 min at room temperature in the dark. The absorbance was measured at 734 nm using a microplate reader.

#### 4.3.2. DPPH Radical Scavenging Activity

The extract (35 µL) was added to 250 µL of methanolic DPPH solution (80 µM) and incubated for 30 min at room temperature in the dark, after which the absorbance was measured at 515 nm.

#### 4.3.3. Ferric-Reducing Antioxidant Power (FRAP) Assay

The FRAP reagent was prepared by mixing acetate buffer (0.3 M, pH 3.6), 2,4,6-tris(2-pyridyl)-s-triazine (10 mM in 40 mM HCl), and FeCl_3_ solution (20 mM in water) at a 10:1:1 (*v*/*v*/*v*) ratio. For the assay, 25 µL of extract was mixed with 175 µL of the FRAP reagent, incubated at room temperature in the dark for 30 min, and the absorbance was measured at 593 nm.

### 4.4. Antimicrobial Activity

#### 4.4.1. Sample Preparation

Prior to analysis, the EECM was centrifuged at 12,000 rpm for 10 min. The resulting clear supernatant was collected and used for antimicrobial and antifungal assays

#### 4.4.2. Microorganisms

The following bacterial, fungus and yeast strains were used for minimum inhibitory concentration (MIC) determination (Table 4).

#### 4.4.3. Minimum Inhibitory Concentration (MIC) Determination

**Figure 11:**
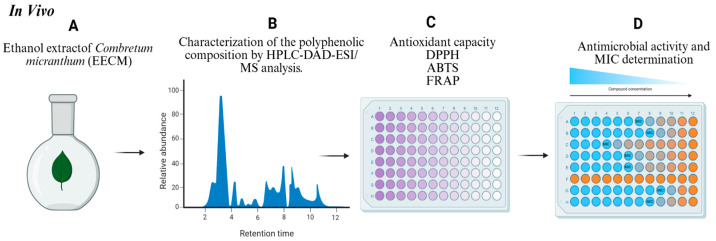
Schematic representation of the in vitro experimental protocol: (**A**) preparation of the ethanolic extract of *Combretum micranthum* (EECM); (**B**) HPLC-DAD-ESI/MS analysis to characterize the polyphenolic composition of the extract and identify and quantify the main bioactive compounds; (**C**) evaluation of antioxidant activity using ABTS, DPPH, and FRAP assays; and (**D**) assessment of antimicrobial activity (Gram-positive and Gram-negative bacteria, filamentous fungi, and yeasts) with determination of the minimum inhibitory concentration (MIC).

Bacterial and yeast cultures were first activated by incubation in their respective broth media at 37 °C for 24 h. Bacterial inocula were standardized to 0.5 McFarland (1.5 × 10^8^ CFU/mL), while fungal spore suspensions of Aspergillus niger were prepared at 10^6^ spores/mL in 0.1% Tween 80. The MIC of the extract was evaluated using a resazurin-based microtiter plate assay. In each well of a 96-well plate, 100 µL of sterile medium and 100 µL of extract were dispensed, followed by twelve 2-fold serial dilutions. Subsequently, 10 µL of inoculum was added (1.5 × 10^5^ CFU/mL for bacteria and 10^6^ CFU/mL for fungi and yeasts). Gentamicin (0.4 mg/mL) and ketoconazole (8.5 mg/mL) were employed as positive controls for bacterial and fungal strains, respectively, while sterile water served as the negative control. Plates were incubated at 37 °C for bacteria (20–22 h) and at 30 °C for fungi and yeasts (20–48 h). Following incubation, 20 µL of resazurin solution (0.2 mg/mL) was added to each well and incubated for an additional 2 h. MIC values were defined as the lowest extract concentration that prevented the resazurin color change from blue to pink, indicating complete inhibition of microbial growth [64]. The in vitro study procedure is illustrated in Figure 11.

### 4.5. In Vivo Study

#### 4.5.1. Animals

C57BL/6 mice (male and female), aged 6–9 weeks (weighing approximately 21 g for males and 16 g for females), were sourced from the Institutul Național de Cercetare-Dezvoltare în Domeniul Patologiei și Științelor Biomedicale “Victor Babeș” Animals were housed in plastic cages under controlled conditions (23–25 °C, 55 ± 10% humidity, and 12 h light/dark cycle) with free access to food and water. Following a 14-day acclimatization period, the experiments complied with the European Directive 2010/63/EU and Romanian Law 43/2014. Ethical approval was granted by the Ethics Committee of the University of Agronomic Sciences and Veterinary Medicine, Cluj-Napoca, Romania (approval no. 478/22.11.2024). An ethical probation was also granted by the National Veterinary and Food Safety Authority (No. 426 dated 14 February 2025).

#### 4.5.2. Dextran Sodium Sulfate (DSS)-Induced Acute Colitis Model

The C57BL/6 mice were randomly divided into four groups: control; 3% DSS; 3% DSS + 100 mg/kg EECM; and 3% DSS + 200 mg/kg EECM (*n* = 10 per group consisting of five males and five females). The control group (non-colitic group) received distilled water, while the other three groups were administered 3% DSS for 7 days to induce colitis. Among these, the 3% DSS group served as the untreated colitic group, while the 3% DSS + 100 mg/kg EECM and 3% DSS + 200 mg/kg EECM groups were administered the ethanolic extract of *Combretum micranthum* G. Don at respective doses of 100 and 200 mg/kg concurrently with colitis induction by 3% DSS for 7 days [26,27]. Animal body weight, presence of gross blood in the feces, and stool consistency were evaluated daily for each mouse by an observer unaware of the treatment. These parameters were assigned a score according to the criteria proposed previously and used to calculate the daily average (DAI) [18].

#### 4.5.3. Evaluation of Disease Activity

In the DSS-induced acute colitis model, body weight, stool consistency, and presence of blood in the feces were recorded daily for each mouse using fresh stool samples. DAI was calculated using scores for body weight loss (0 = none; 1 = 1–5%; 2 = 5–10%; 3 ≥ 10%), stool consistency (0 = normal; 1 = slightly loose stools; 2 = loose stools; 3= watery diarrhea), and the presence of blood in stools (0, none; 1, slightly bloody; 2, bloody; 3, gross bleeding), based on a previous scoring system described by Zhou Y et al. [27]. Briefly, DAI = [(weight loss score) + (stool consistency score) + (blood score)]/3 (Table 5).

#### 4.5.4. Anesthesia and Euthanasia Procedures

Animals were placed in a sealed induction chamber and administered a high dose of isoflurane via inhalation to achieve deep sedation and ensure consistent exposure. Once deep anesthesia was confirmed by the absence of reflex responses, cervical dislocation was performed as a secondary method to ensure euthanasia. This process was carried out in full compliance with Directive 2010/63/EU of the European Parliament and of the Council of 22 September 2010, which pertains to the protection of animals used for scientific purposes, thereby adhering to established ethical guidelines [68,69].

#### 4.5.5. Blood and Organ Sampling

Blood samples were drawn into ethylenediaminetetraacetic acid (EDTA)-containing tubes for hematological evaluation and into heparinized tubes for biochemical analysis. The colon was collected and fixed in 10% formalin for histological examination.

#### 4.5.6. Hematological and Biochemical Analyses

Hematological parameters were assessed using an automated hematology analyzer (Diatron Abacus Junior 5). The parameters measured included: white blood cells (WBCs), Neutrophils (NEUs), Lymphocytes (LYMs), Monocytes (MONs), Eosinophil (ESO), Basophils (BASs), Red Blood Cells (RBCs), Hemoglobin (HGB), Hematocrit (HCT), Mean Corpuscular Volume (MCV), Mean Corpuscular Hemoglobin (MCH), Red Cell Distribution Width—Coefficient of Variation (RDW-CV), Red Cell Distribution Width—Standard Deviation (RDWC-DS), Mean Platelet Volume (MPV), Platelet Distribution Width (PDW), and Plateletcrit (PCT).

Biochemical analysis was conducted using an automated chemistry analyzer (Scil—Element RC). The parameters measured were albumin (ALB), total proteins (TP), globulins (GLOB), albumin/globulin ratio (A/G), total bilirubin (TB), alanine transaminase (ALT), alkaline phosphatase (ALP), amylase (AMY), creatinine (CREA), urea (UREA), glucose (GLU), serum calcium (Ca), phosphate (PHOS), potassium (K), and sodium (Na).

#### 4.5.7. Histopathological Analysis

The harvested tissues were fixed in 10% formalin for 48 h. After fixation, the samples were dehydrated in ascending ethanol concentrations and clarified, which was achieved by immersion in ethyl alcohol baths with increasing concentration, respectively, in xylene baths. After clarifying the samples, the samples’ infiltration with paraffin followed, was carried out at 58 °C for 5 h, using low-melting-point paraffin. After this, thin sections of 2 µm were obtained from the paraffin blocks using the rotary microtome. According to a routine protocol, they were later stained with hematoxylin–eosin (H&E) according to a standard protocol. Histological samples were examined under an Olympus BX51 microscope and the bright field images were obtained with an Olympus SP350 digital camera and processed using the Olympus Cell Sens software (version 2.1). The histological score was calculated on the basis of the degrees of colon inflammation; the morphological criteria of grading used were those previously recommended by [14] and described by [70].

The microscopic scoring criteria for full-thickness distal colon sections involve the histological evaluation of several structures: the mucosal epithelium and lamina propria, the crypts, the submucosa, and the muscular layer. For the mucosal epithelium and lamina propria, ulceration is scored from 0 to 4 according to its extent: none (0), mild and superficial (0–25%) (1), moderate (25–50%) (2), severe (50–75%) (3), or extensive, involving the full thickness (more than 75%) (4). The presence of polymorphonuclear cell infiltration, mononuclear cell infiltration associated with fibrosis, as well as edema and dilation of lacteals, are also evaluated. At the level of the crypts, mitotic activity is graded according to its location: limited to the lower third (0), mild in the microscopic scoring criteria for full-thickness distal colon sections involve the histological evaluation of several structures: the mucosal epithelium and lamina propria, the crypts, the submucosa, and the muscular layer. For the mucosal epithelium and lamina propria, ulceration is scored from 0 to 4 according to its extent: none (0), mild and superficial (0–25%) (1), moderate (25–50%) (2), severe (50–75%) (3), or extensive, involving the full thickness (more than 75%) (4). The presence of polymorphonuclear cell infiltration, mononuclear cell infiltration associated with fibrosis, as well as edema and dilation of lacteals, are also evaluated. At the level of the crypts, mitotic activity is graded according to its location: limited to the lower third (0), mild in the middle third (1), moderate in the middle third (2), or extending to the upper third (3). Crypt dilation and goblet cell depletion are also considered. In the submucosa, the analysis includes the presence of polymorphonuclear and mononuclear cell infiltrates, as well as edema. Finally, the muscular layer is evaluated for the presence of polymorphonuclear and mononuclear cell infiltrates, edema, and possible infiltration into the serosa. Apart from the classification system described above, microscopic changes were also graded for severity using a standard scale from 0 to 4, where 0 = no significant change, 1 = minimal, 2 = mild, 3 = moderate, and 4 = severe.

#### 4.5.8. Statistical Analysis

**Figure 12:**
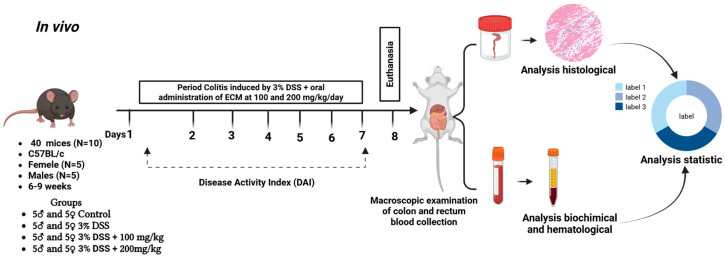
Schematic of the in vivo protocol: male and female C57BL/6 mice received 3% DSS to induce acute colitis and were then concurrently treated with EECM at 100 and 200 mg/kg orally for 7 days. The progression of colitis was monitored daily using the Disease Activity Index (DAI), and at the end of the experiment, macroscopic, histological, hematological, and biochemical analyses were performed to assess colon integrity and the systemic protective effect of EECM.

Data are presented as mean ± standard deviation (SD) for ten animals per group, as appropriate. Statistical analysis was per-formed using Graph Pad Prism 10 (San Diego, CA, USA). Group comparisons were conducted using One-way ANOVA followed by Tukey’s multiple comparisons post hoc test. A *p*-value < 0.05 was considered statistically significant The in vivo study procedure is illustrated in Figure 12.

## 5. Conclusions

This study highlights the significant therapeutic effect of the ethanolic extract of *Combretum micranthum* (EECM) in alleviating DSS-induced colitis in C57BL/6 mice, emphasizing its strong potential as a natural agent against intestinal inflammation. This activity is attributed to its richness in bioactive phenolic compounds and its anti-inflammatory, antioxidant, and antibacterial properties. The extract’s efficacy is demonstrated by a reduction in colonic tissue damage, preservation of histological architecture, and favorable modulation of biochemical and hematological markers.

Future research should focus on the long-term effects of EECM in the treatment of chronic colitis. These findings also highlight the potential of EECM as a basis for developing phytomedicines or dietary supplements aimed at preventing or treating inflammatory bowel diseases, while modulating the gut microbiota and minimizing the side effects of conventional therapies. Overall, these results pave the way for further preclinical and clinical studies to confirm its efficacy and safety.

## Figures and Tables

**Table 1:** Values of the Ethanolic Extract of *Combretum micranthum* determined by DPPH, ABTS and FRAP Radical Scavenging Assays (mmol TE/g of sample).

DPPH	ABTS	FRAP
135.86 ± 2.35	233.72 ± 2.81	428.86 ± 145.09

**Table 2:** Minimum inhibitory concentrations (MIC, µg/mL) of the ethanolic extract of *Combretum micranthum* against Gram-negative and Gram-positive bacteria.

Sample	Gram-Negative Bacteria (−)		Gram-Positive Bacteria (+)				
	*E. coli*	*S. enterica*	*P. aeruginosa*	*Bacillus subtillis*	*S. pyogenes*	*S. aureus*	*Enterococcus fecalis*
*Combretum micranthum*	n.b.	1250	n.b.	12,500	12,500	25,000	n.b.
Water (C^−^)	n.b.	n.b.	n.b.	n.b.	n.b.	n.b.	n.b.
Gentamicin (C^+^)	6.25	1.653	0.391	1.563	6.25	0.391	50

n.b.—no bioactivity.

**Table 3:** Minimum inhibitory concentrations (MIC, µg/mL) of the ethanolic extract of *Combretum micranthum* against yeast and fungi.

Sample	Yeasts			Fungi
	*Sacharomyces cerevisiae* var. *diastaticus*	*Candida parapsilosis*	*Candida albicans*	*Aspergillus* *brasiliensis*
*Combretum micranthum*	n.b.	n.b.	n.b.	n.b.
Water (C^−^)	n.b.	n.b.	n.b.	n.b.
Ketoconaloze (C^+^)	1062.5	2125	531.25	312.5

n.b.—no bioactivity.

**Table 4:** Microbial strains used for antimicrobial testing and their culture media.

Type	Strain ID/ATCC	Medium Used
Gram-positive bacteria	*Staphylococcus aureus* ATCC 25923	Tryptic Soy Broth (TSB)
	*Bacillus subtilis* ATCC 1177	Nutrient Broth (NB)
	*Enterococcus faecalis* ATCC 29212	Brain Heart Infusion (BHI)
	*Streptococcus pyogenes* ATCC 12344	
Gram-negative bacteria	*Escherichia coli* ATCC 25922	TSB
	*Pseudomonas aeruginosa* ATCC 27853	NB
	*Salmonella enterica* (typhimurium) ATCC 14028	
Filamentous fungus	*Aspergillus niger* ATCC 6275	Potato Dextrose Agar (PDA)
Yeasts	*Sacharomyces cerevisiae* var. *diastaticus* M29	Yeast Medium (YM)
	*Candida. parapsilosis* ATCC 22019	
	*Candida albicans* DSMZ 1387	

**Table 5:** Disease Activity Index (DAI) Scores.

Weight Loss%	Stool Traits	Fecal Occult Blood/Gross Blood in the Stool	Scores
0	normal	normal	0
1–5	loose	occult blood weakly positive	1
5–10	soft stool	occult blood strongly positive	2
10–15	loose stool	gross blood in the stool	3
>15	diarrhea	massive bleeding in the stool	4

DAI score = (body weight loss score + stool consistency score + occult blood score)/3.

## Data Availability

The original contributions presented in this study are included in the article/Supplementary Materials. Further inquiries can be directed to the corresponding author.

## Supplementary Materials

The following supporting information can be downloaded at: https://www.mdpi.com/article/10.3390/ijms262311299/s1.

## Abbreviations

A/G	Albumin/Globulin ratio
ABTS	2,2′-Azino-bis (3-ethylbenzothiazoline-6-sulfonic acid)
ALB	Albumin
ALP	Alkaline Phosphatase
AMY	Amylase
BAS	Basophils
BHI	Brain Heart Infusion
Ca	Calcium
COX-2	Cyclooxygenase-2
CREA	Creatinine
DAI	Disease Activity Index
DPPH	2,2-diphényl-1-picrylhydrazyl
DSS	Dextran Sulfate Sodium
EECM	Ethanolic Extract of *Combretum micranthum*
ESO	Eosinophils
FRAP	Ferric Reducing Antioxidant Power
GLU	Glucose
GLOB	Globulins
HCT	Hematocrit
HGB	Hemoglobin
HO-1	Heme Oxygenase-1
HPLC-DAD-ESI-MS	High-Performance Liquid Chromatography—Diode Array Detector—Electrospray Ionization—Mass Spectrometry
IBD	Inflammatory Bowel Diseases
IL-1β, IL-6	Interleukins 1 beta and 6
K	Potassium
LYM	Lymphocytes
MCV	Mean Corpuscular Volume
MCH	Mean Corpuscular Hemoglobin
MPV	Mean Platelet Volume
MON	Monocytes
NA	Sodium
NEU	Neutrophils
NO	Nitric Oxide
NB	Nutrient Broth
PDA	Potato Dextrose Agar
PDW	Platelet Distribution Width
PCT	Plateletcrit
PHOS	Phosphate
RBC	Red Blood Cells
RDW-CV	Red Cell Distribution Width—Coefficient of Variation
RDW-SD	Red Cell Distribution Width—Standard Deviation
ROS	Reactive Oxygen Species
TB	Total Bilirubin
TP	Total Proteins
TNF-α	Tumor Necrosis Factor-alpha
TLR	Toll-Like Receptor
TSB	Tryptic Soy Broth
UREA	Urea
WBC	White Blood Cells
YM	Yeast Medium
OMVs	bacterial outer membrane vesicles
LPS	lipopolysaccharides
