# Supplementary material for: Ethanolic Extract of Kinkeliba (Combretum micranthum), Rich in Phenolic Compounds Mitigates DSS-Induced Ulcerative Colitis in C57BL/6 Mice via Antioxidation and Microbiota Regulation
Source: Int J Mol Sci. 2025 Nov 22;26(23):11299. doi: 10.3390/ijms262311299 (PMC12692256; doi:10.3390/ijms262311299)
Supplement: Supplementary file 1 [file ijms-26-11299-s001.zip › ijms-3947358-supplementary.pdf]

Table S1: The content of phenolic compounds in ethanolic extracts of C.micranthum leaves by HPLC-DAD-ESI-MS (mg/g of extract).

	Phenolic Compounds	Subclass	R_t (min)	λmax (nm)	$[M+H]^+$ (m/z)	Extract (mg/g)
**1**	Gallic acid	Hydroxybenzoic acid	4.70	275	171	4.80
**2**	Protocatechuic acid	Hydroxybenzoic acid	9.01	280	155	14.14
**3**	1,6-Digalloyl-glucose	Gallotannin	13.16	280	485	5.82
**4**	Ellagic acid-arabinoside	Hydroxybenzoic acid	13.93	270, 360	435	14.04
**5**	Ellagic acid-glucoside	Hydroxybenzoic acid	14.43	270, 360	465	8.08
**6**	Sanguiin H-4	Ellagitannin	15.14	270, 360	635	102.56
**7**	Corilagin	Ellagitannin	16.22	270, 360	635	63.29
**8**	Ellagic acid	Hydroxybenzoic acid	16.63	270, 360	303	12.10
**9**	Combretastatin B1	Stilbene	22.69	275	335	68.71
	**Total phenolics** (mg/g)					**293.54**

Table S2: Hematological parameters of males and females' mice in C57BL/6

Hematological parameters	Control ♂	3 % DSS ♂	3 % DSS + 100 mg/kg ♂	3 % DSS + 200mg/kg♂	Control ♀	3 % DSS♀	3 % DSS + 100 mg/kg ♀	3 % DSS + 200mg/kg♀
WBC (10^9/L)	2.26 ± 0.1	17,34 ± 0.9 ###	2.29± 0.2 €€€	2.27± 0.1 ***	4.29 ± 0.3	39,43 ± 2.4 ###	4.62 ± 0.4 €€€	4.28 ± 0.4 ***
NEU (10^9/L)	0.29 ± 0.01	1.46 ± 0.02 ##	0.35 ± 0.01€€	0 .37± 0.01 **	0.50 ± 0	7.93 ± 0.1 ###	3.85 ± 0.6 €€€	1 .59 ± 0.1 ***
LYM (10^9/L)	1.82 ± 0.01	13,75±1.01###	2.12 ± 0.04 €€€	2.15± 0.03 ***	3.79 ± 0.2	30,20 ± 1.2 ###	5.36 ± 0.9 €€€	4.02 ± 0.8 ***
MON (10^9/L)	0.4 ± 0	2.10 ± 0 ##	0.3 ± 0 €€	0.3 ± 0 **	0.9 ± 0	1.10 ± 0	0.18 ± 0	0.16 ± 0
EOS (10^9/L)	0.10 ± 0	0.12 ± 0	0.11 ± 0	0.11 ± 0	0.12 ± 0	0.11 ± 0	0.12 ± 0	0.12 ± 0
BAS (10^9/L)	0 ± 0	0.02 ± 0	0 ± 0	0 ± 0	0 ± 0	0.02 ± 0	0 ± 0	0 ± 0
RBC (10^{12}/L)	10.13 ± 0.9	9.93 ± 0.8	10.16 ± 0.9	10.14 ± 1.1	9.13 ± 2.1	5.47 ± 2.2 ###	9.01 ± 1.1 €€€	9.15 ± 1.9 ***
HGB (g/L)	149 ± 7.1	100 ± 3.4 ###	150 ± 5.2 €€€	153 ± 3.3 ***	149 ± 4.9	84 ± 4.6 ###	147 ± 5.5 €€€	144 ± 6 ***
HCT (%)	0.484 ± 0.03	0.394 ± 0.01	0.485 ± 0.01	0.488 ± 0.01	0.446 ± 0.1	0.444 ± 0.1	0.448 ± 0.1	0.487± 0.1
MCV (fL)	47.8 ± 3	46.7 ± 2	48.0 ± 2	48.4 ± 2	47.8 ± 3.1	48.9 ± 5.1	47.7 ± 3.3	47.5 ± 4.2
MCH (pg)	14.8 ± 0.9	14.8 ± 3.1	14.3 ± 3.6	14.8 ± 3.4	16.3 ± 3.2	15.4 ± 2.1	16.2 ± 3.6	16.1 ± 4.3
RDW-CV	0.138 ± 0.01	0.139 ± 0.01	0.138 ± 0.01	0.139 ± 0.01	0.139 ± 0	0.140 ± 0	0.142 ± 0	0.140 ± 0
RDWC-SD (fL)	24.6 ± 4	25.4 ± 5.5	24.6 ± 5.2	24.3 ± 3.5	23.8 ± 3.2	23.9 ± 2.2	23.8 ± 2.4	23.7 ± 2.2
PLT (10^9/L)	867 ± 11.2	600 ± 10.3###	870 ± 12.2 €€€	875 ± 13.2 ***	484 ± 13.5	836 ± 15 ###	489 ± 10 €€€	488 ± 12 ***
MPV (fL)	5.7 ± 0.08	6 ± 0.06	5.7 ± 0.07	5.7 ± 0.07	5.5 ± 1.1	5.6 ± 0.9	5.7 ± 1.2	5.6 ± 0.8
PDW	15.5 ± 1	15.7 ± 2.1	15.3 ± 3.1	15.2 ± 2.9	15.2 ± 2.2	15.7 ± 2.2	15.3 ± 2.3	15.3 ± 1.2
PCT (Ml/l)	4.94 ± 0.9	4.80 ± 1.1	4.95 ± 1.1	4.96± 1.3	2.66 ± 0.2	2.02 ± 0.9	2.73 ± 0.1	2.74± 0.2

Table S3: Biochemical parameters of males and females' mice in C57BL/6

Hematological parameters	Control ♂	3 % DSS ♂	3 % DSS + 100 mg/kg ♂	3 % DSS + 200mg/kg♂	Control ♀	3 % DSS♀	3 % DSS + 100 mg/kg ♀	3 % DSS + 200mg/kg♀
**ALB** (g/dl)	2.9 ± 0.11	2.8± 0.19	2.8 ± 0.10	2.8 ± 0.15	2.8 ± 0.15	2.9± 0.10	2.7 ± 0.14	2.7 ± 0.10
**TP** (g/dl)	4.6 ± 0.10	4.5 ± 0.28	4.6 ± 0.17	4.6 ± 0.24	4.6 ± 0.12	4.6± 0.40	4.5 ± 0.11	4.6 ± 0.20
**GLOB** (g/dl)	2.0 ± 0.19	2.1 ± 0.17	2.0 ± 0.14	2.1 ± 0.17	2.0 ± 0.11	2.1 ± 0.15	1.9 ± 0.10	2.0± 0.12
**A/G**	1.31± 0.01	1.29 ± 0.02	1.31± 0.01	1.33 ± 0.02	1.45± 0.01	1.42 ± 0.01	1.48± 0.20	1.42 ± 0.03
**TB** (g/dl)	0.4 ± 0.09	0.3± 0.04	0.3 ± 0.07	0.4 ± 0.05	0 ± 0	0.1± 0	0.1 ± 0	0.1 ± 0
**ALT** (u/l)	36 ± 1.8	28 ± 2.5 ###	35 ± 1.5€€€	34 ± 1.1***	38 ± 2.5	22 ± 1.5 ###	36 ± 1.5	38 ± 2.5
**ALP** (u/l)	92± 2	43 ± 2 ###	84± 1 €€€	72± 1.5 ***	83± 5	33 ± 3.5 ###	82± 1 €€€	85± 2.6 ***
**AMY** (u/l)	1807 ± 11.75	3374 ± 11.10 ###	1971 ± 15.15 €€	1937 ± 13.15 **	1643 ± 10	2212 ± 11.10 ###	1654 ± 10 €€	1639 ± 14.16 **
**CREA**(mg/dl)	0.2 ± 0	0.3 ± 0	0.2 ± 0	0.2 ± 0	0.1 ± 0	0.1 ± 0	0.1 ± 0	0.1 ± 0
**UREA**(mg/dl)	57.83 ± 2. 5	136.34 ± 2.1 ###	56.88 ± 2.2€€€	56.03 ± 3***	57.70 ± 4. 7	114.70 ± 5 ###	63.23 ± 3.1€€	60.90 ± 5**
**GLU** (mg/dl)	91.02 ± 1	124.41 ± 2 ###	94.91 ± 2€€€	94.42 ± 1.5***	91.02 ± 5.6	118.21 ± 5.5	90.10 ± 4.4	89.42 ± 4.5
**Ca** (mg/dl)	6.63 ± 1.2	6.58 ± 0.9	6.84 ± 0.5	6.53 ± 0.4	6.93 ± 0.9	6.44 ± 0.5	6.31 ± 0.6	6.51 ± 0.8
**PHOS**(mg/dl)	9.52 ± 2.1	9.58 ± 1.9	9.54 ± 1.5	9.26 ± 2	9.84 ± 1.5	9.85 ± 1.5 #	9.24 ± 2.5	10.26 ± 1.5
**K^+** (mmol/L)	8.0 ± 2	8.0 ± 2.3	8.12 ± 1.9	8.02 ± 1.5	8 ± 2	8 ± 2.3	8.5 ± 1.5	8. ± 2.5
**Na^+** (mmol/L)	145.8 ± 8.3	146.48 ± 5.5	146.20 ± 6.1	146.5 ± 4.4	147.2 ± 5.3	147.1 ± 4.2	148.2 ± 5.5	147.8 ± 4.5

Table S4: Summary of Intestinal Histopathological Scores in Mice Induced by DSS + EECM Administration

Group	Animal	Mucosal epithelium and lamina propria				Crypts			Submucosa			Muscular layer			
		Erosions/Ulceration	PMN Infiltrate	MC infiltrate and fibrosis	Oedema/dilatation of lacteals	Mitotic activity	Dilatation	Goblet cell depletion	PMN Infiltrate	MC infiltrate	Oedema	PMN Infiltrate	MC infiltrate	Oedema	Infiltration in the serosa
**Control ♂**	**Average /animal=**	**0**	**0,2**	**0,2**	**0**	**0**	**0**	**0**	**0**	**0**	**0**	**0**	**0**	**0**	**0**
**Control ♀**	**Average /animal=**	**0**	**0,2**	**0,2**	**0**	**0**	**0**	**0**	**0**	**0**	**0**	**0**	**0**	**0**	**0**
**3% DSS ♂**	**Average /animal=**	**2,2**	**2,2**	**2,6**	**1**	**1,6**	**1,4**	**1**	**2,4**	**2**	**1**	**1**	**0,8**	**0,8**	**1,2**
**3% DSS ♀**	**Average /animal=**	**1,6**	**2,6**	**2,6**	**0,6**	**1,8**	**1,8**	**1,6**	**2,4**	**2,4**	**2,2**	**1,2**	**1,2**	**1,2**	**1,2**
**100 mg/kg ♂**	**Average /animal=**	**0**	**1**	**2**	**0,8**	**1,2**	**0,4**	**0,6**	**1**	**1**	**0,8**	**0,2**	**0,4**	**0,2**	**0,2**
**100 mg/kg ♀**	**Average /animal=**	**1,2**	**1**	**1,6**	**0**	**0,6**	**0,4**	**0,4**	**1,2**	**1**	**1,8**	**0**	**0**	**0**	**0,2**
**200 mg/kg ♂**	**Average /animal=**	**1**	**0,8**	**1,8**	**0,8**	**1**	**0,4**	**0,6**	**0,8**	**0,8**	**0,6**	**0**	**0,2**	**0**	**0,2**
**200 mg/kg ♀**	**Average /animal=**	**1**	**0,8**	**1,6**	**0,8**	**0,4**	**0**	**0**	**0,8**	**0,8**	**0,8**	**0,2**	**0,2**	**0,6**	**0,2**
